# Characterization of DNA Gyrase Activity and Elucidation of the Impact of Amino Acid Substitution in GyrA on Fluoroquinolone Resistance in Mycobacterium avium

**DOI:** 10.1128/spectrum.05088-22

**Published:** 2023-04-17

**Authors:** Jeewan Thapa, Joseph Yamweka Chizimu, Soyoka Kitamura, Mwangala Lonah Akapelwa, Pondpan Suwanthada, Nami Miura, Jirachaya Toyting, Tomoyasu Nishimura, Naoki Hasegawa, Yukiko Nishiuchi, Stephen V. Gordon, Chie Nakajima, Yasuhiko Suzuki

**Affiliations:** a Division of Bioresources, International Institute for Zoonosis Control, Hokkaido University, Sapporo, Japan; b Zambian National Public Health Institute, Ministry of Health, Lusaka, Zambia; c Department of Medical Laboratory Science, Faculty of Health Sciences, Hokkaido University, Sapporo, Japan; d Keio University Health Center, Tokyo, Japan; e Department of Infectious Diseases, Keio University School of Medicine, Tokyo, Japan; f Toneyama Institute for Tuberculosis Research, Osaka City University Medical School, Osaka, Japan; g Office of Academic Research and Industry-Government Collaboration, Section of Microbial Genomics and Ecology, Hiroshima University, Higashi-Hiroshima, Japan; h School of Veterinary Medicine, University College Dublin, Dublin, Ireland; i International Collaboration Unit, International Institute for Zoonosis Control, Hokkaido University, Sapporo, Japan; j Institute for Vaccine Research and Development, Hokkaido University, Sapporo, Japan; Johns Hopkins University School of Medicine

**Keywords:** *Mycobacterium avium*, DNA gyrase, fluoroquinolone resistance, supercoiling assay, minimum inhibitory concentration

## Abstract

Mycobacterium avium, a member of the M. avium complex (MAC), is the major pathogen contributing to nontuberculous mycobacteria (NTM) infections worldwide. Fluoroquinolones (FQs) are recommended for the treatment of macrolide-resistant MACs. The association of FQ resistance and mutations in the quinolone resistance-determining region (QRDR) of *gyrA* of M. avium is not yet clearly understood, as many FQ-resistant clinical M. avium isolates do not have such mutations. This study aimed to elucidate the role of amino acid substitution in the QRDR of M. avium GyrA in the development of FQ resistance. We found four clinical M. avium subsp. *hominissuis* isolates with Asp-to-Gly change at position 95 (Asp95Gly) and Asp95Tyr mutations in *gyrA* that were highly resistant to FQs and had 2- to 32-fold-higher MICs than the wild-type (WT) isolates. To clarify the contribution of amino acid substitutions to FQ resistance, we produced recombinant WT GyrA, GyrB, and four GyrA mutant proteins (Ala91Val, Asp95Ala, Asp95Gly, and Asp95Tyr) to elucidate their potential role in FQ resistance, using them to perform FQ-inhibited DNA supercoiling assays. While all the mutant GyrAs contributed to the higher (1.3- to 35.6-fold) FQ 50% inhibitory concentration (IC_50_) than the WT, Asp95Tyr was the most resistant mutant, with an IC_50_ 15- to 35.6-higher than that of the WT, followed by the Asp95Gly mutant, with an IC_50_ 12.5- to 17.6-fold higher than that of the WT, indicating that these amino acid substitutions significantly reduced the inhibitory activity of FQs. Our results showed that amino acid substitutions in the *gyrA* of M. avium contribute to FQ resistance.

**IMPORTANCE** The emergence of fluoroquinolone (FQ) resistance has further compounded the control of emerging Mycobacterium avium-associated nontuberculous mycobacteria infections worldwide. For M. avium, the association of FQ resistance and mutations in the quinolone resistance-determining region (QRDR) of *gyrA* is not yet clearly understood. Here, we report that four clinical M. avium isolates with a mutation in the QRDR of *gyrA* were highly resistant to FQs. We further clarified the impact of mutations in the QRDR of GyrA proteins by performing *in vitro* FQ-inhibited DNA supercoiling assays. These results confirmed that, like in Mycobacterium tuberculosis, mutations in the QRDR of *gyrA* also strongly contribute to FQ resistance in M. avium. Since many FQ-resistant M. avium isolates do have these mutations, the detailed molecular mechanism of FQ resistance in M. avium needs further exploration.

## INTRODUCTION

Nontuberculous mycobacteria (NTM) infections are an emerging public health problem worldwide (1). Of the approximately 200 NTM species recognized to date, the majority are environmental bacteria that are nonpathogenic to humans and animals (2). In cases of NTM infections, lung disease is the most common clinical manifestation, and the major causative agent is Mycobacterium avium complex (MAC) (3). New cases of NTM infections are increasing worldwide, including in the United States and Japan (4, 5). While NTM incidence has surpassed that of tuberculosis in developed countries, its incidence in developing countries may be underestimated because of inaccurate diagnosis (6). Among MACs, M. avium and Mycobacterium intracellulare are the major pathogens of clinical significance (7). In Japan, M. avium was found to be the most common species of MAC and the highest contributor to NTM lung disease (4, 7). The widespread distribution of M. avium in Japan has been associated with particular environments, such as bathrooms (8).

Due to an increasing number of cases of macrolide-resistant MAC infections, a situation analogous to that of multidrug-resistant tuberculosis (9, 10), fluoroquinolones (FQs) are recommended for treatment of macrolide-resistant MAC (10, 11), supported by drug sensitivity testing results. The increasing usage of FQs against MAC has led to cases of clinical resistance (12–15). The FQs interfere with DNA gyrase, an essential bacterial enzyme in DNA replication and transcription. DNA gyrase is a hetero-tetrameric enzyme, consisting of two subunits of GyrA and two subunits of GyrB (16). Mutations in the quinolone resistance-determining regions (QRDR) of *gyrA* and *gyrB* resulting from amino acid substitution in the GyrA and GyrB subunits of DNA gyrase confer FQ resistance (17, 18). The role of these QRDR mutations has been well established in different bacteria, such as Mycobacterium tuberculosis (19–22), Campylobacter jejuni (23), and Salmonella enterica serovar Enteritidis (24), to name a few.

**FIG 1:**
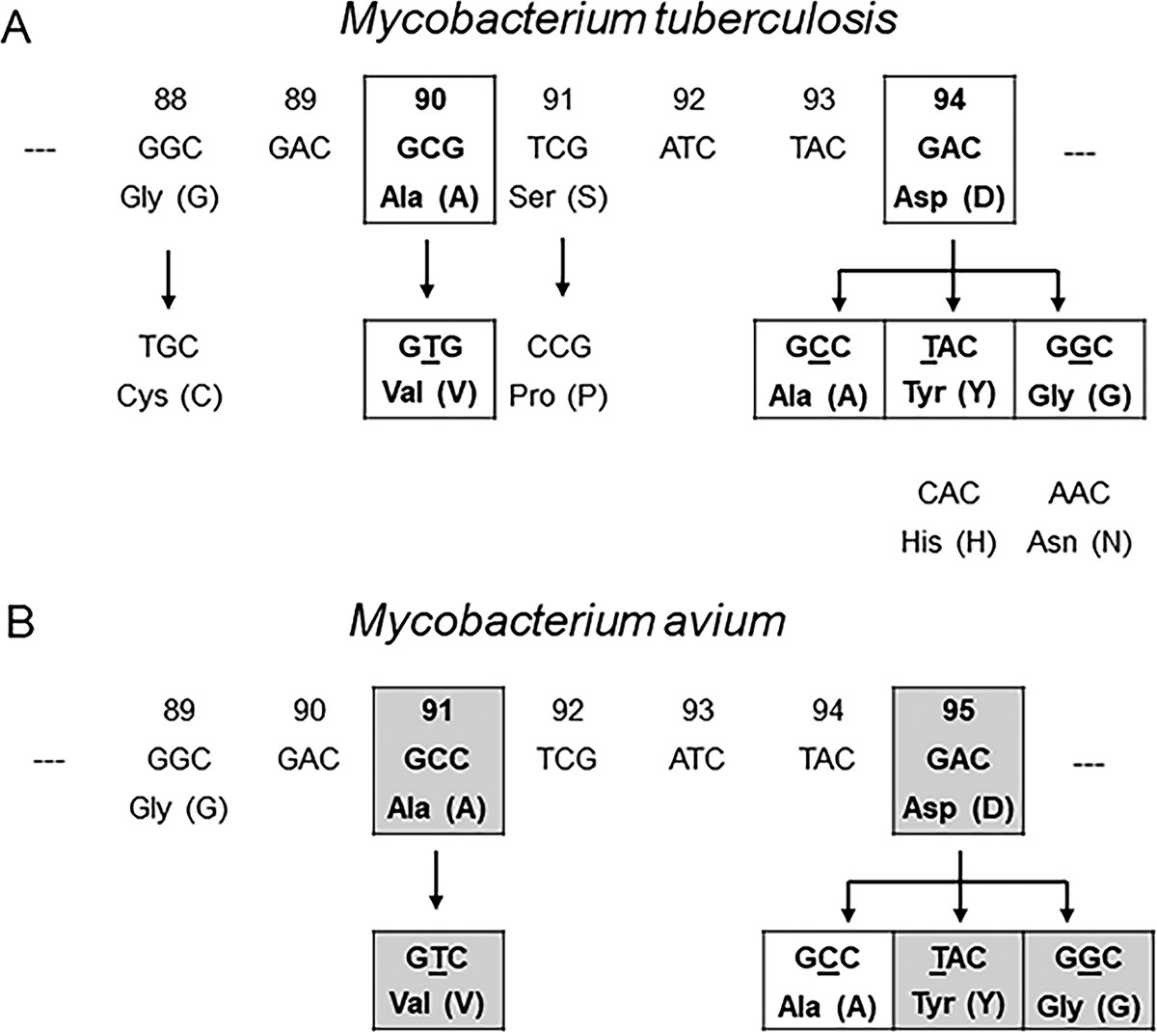
Nucleotide and amino acid substitutions in the QRDR of GyrA in M. tuberculosis and M. avium. The number denotes the corresponding amino acid in GyrA. (A) Nucleotide and amino acid substitutions in the quinolone resistance-determining GyrA region of quinolone-resistant clinical M. tuberculosis isolates obtained from the WHO mutation catalog of 2021 (19). Boxed amino acid substitutions were used to construct mutant M. avium *gyrA*. Mutated bases are underlined. (B) Nucleotide and amino acid substitutions in the quinolone resistance-determining GyrA region of M. avium isolates. The number denotes the GyrA amino acid position, equivalent to the amino acid position in M. tuberculosis. Gray filled boxes indicate an amino acid substitution associated with FQ resistance in a clinical and an *in vitro* study.

Despite the use of quinolones for NTM treatment, the association of mutations in the QRDR of MAC with resistance is not clearly established. Previous studies from Japan and South Korea did not find any mutations in the *gyrA* and *gyrB* genes of moxifloxacin (MOX)-resistant M. avium and M. intracellulare isolates (13, 15). Although a previous study from China reported mutations in *gyrA* and *gyrB* in M. avium isolates, the description of those mutations did not clarify if they were in the QRDR (12). However, a previous study established an *in vitro* ofloxacin-resistant M. avium mutant strain with an Ala91Val mutation in *gyrA* (25). In our ongoing sequence analysis of M. avium subsp. *hominissuis* isolates from Japan, we found four isolates among which two isolates each had an Asp-to-Gly change at position 95 (Asp95Gly) or Asp95Tyr mutation in *gyrA*, motivating us to understand the impact of these mutations on FQ resistance in M. avium. Similarly, a previous study from Brazil reported Ala91Val, Asp95Val, Asp95Gly, and Asp95Asn mutations in *gyrA* in several NTM isolates (26). These results suggested these amino acid substitutions may have been selected due to their impact on FQ resistance in M. avium, thus prompting us to explore the correlation between these amino acid substitutions in DNA gyrase and FQ resistance in M. avium. In M. tuberculosis, up to 90% of FQ-resistant strains harbor mutations in the QRDR of *gyrA* (27). In a recent World Health Organization catalog of mutations in M. tuberculosis complex and their association with drug resistance, amino acid substitutions in GyrA at positions Ala90Val, Asp94Ala, Asp94Gly, and Asp94Tyr (equivalent to Ala91Val, Asp95Ala, Asp95Gly, and Asp95Tyr in M. avium) were the major mutations conferring FQ resistance (Fig. 1) (19, 22, 28). Furthermore, these or equivalent mutations have been validated as contributing to FQ resistance *in vitro* by a DNA gyrase supercoiling assay in various bacteria, such as amino acids Ser83 and Asp87 in Escherichia coli GyrA (18, 29) and Salmonella (24) (equivalent to Ala91 and Asp95 in M. avium GyrA). Amino acids Ala91 and Asp95 in M. avium GyrA (equivalent to Ser83 and Asp87 in Escherichia coli and Ala90 and Asp94 in M. tuberculosis), which lie in the α4 helix of the helix-turn-helix region of GyrA, may have the potential to be commonly mutated in a similar way to the equivalent amino acid positions in E. coli and M. tuberculosis GyrA (30).

Despite the widespread acceptance that FQ resistance in M. tuberculosis mainly develops from mutations in the drug target sites (19), it remains unclear to what extent this association holds true for NTMs, including M. avium. Therefore, the aim of this study was to elucidate the impact of specific FQ-associated amino acid substitutions in the GyrA of M. avium on FQ resistance by characterizing DNA gyrase activity of M. avium.

## RESULTS

### Expression and purification of recombinant M. avium DNA gyrase.

The wild-type (WT) *gyrA* and *gyrB* genes were PCR amplified from the genomic DNA of M. avium subsp. *hominissuis* strain (HP 59), isolated from Hokkaido, Japan (31), and were inserted into a pET29a+ expression vector as described in Materials and Methods. Recombinant WT or mutant GyrA and GyrB subunits were purified as soluble proteins with the expected molecular weights of 93 kDa (GyrA) and 76 kDa (GyrB) by two-step column chromatography. The purity of the expressed proteins was determined to be over 95% by sodium dodecyl sulfate-polyacrylamide gel electrophoresis (SDS-PAGE) (see Fig. S1 in the supplemental material).

### DNA supercoiling activities.

**FIG 2:**
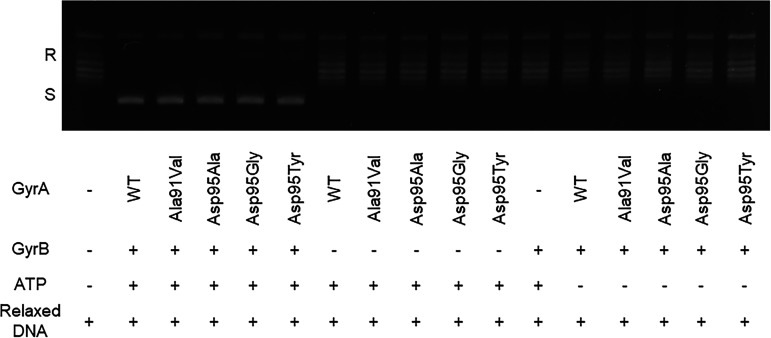
ATP-dependent DNA supercoiling assay. Supercoiling activities of WT-GyrA, Ala91Val GyrA, Asp95Ala GyrA, Asp95Gly GyrA or Asp95Tyr GyrA, and WT-GyrB. A 7.5 nM concentration of each DNA gyrase subunit, 1.5 nM pBR32 relaxed DNA, and 1 mM ATP were used as indicated in the corresponding lanes. DNA supercoiling activity was observed only in the presence of ATP, GyrA, and GyrB subunits. R, relaxed DNA; S, supercoiled DNA.

**FIG 3:**
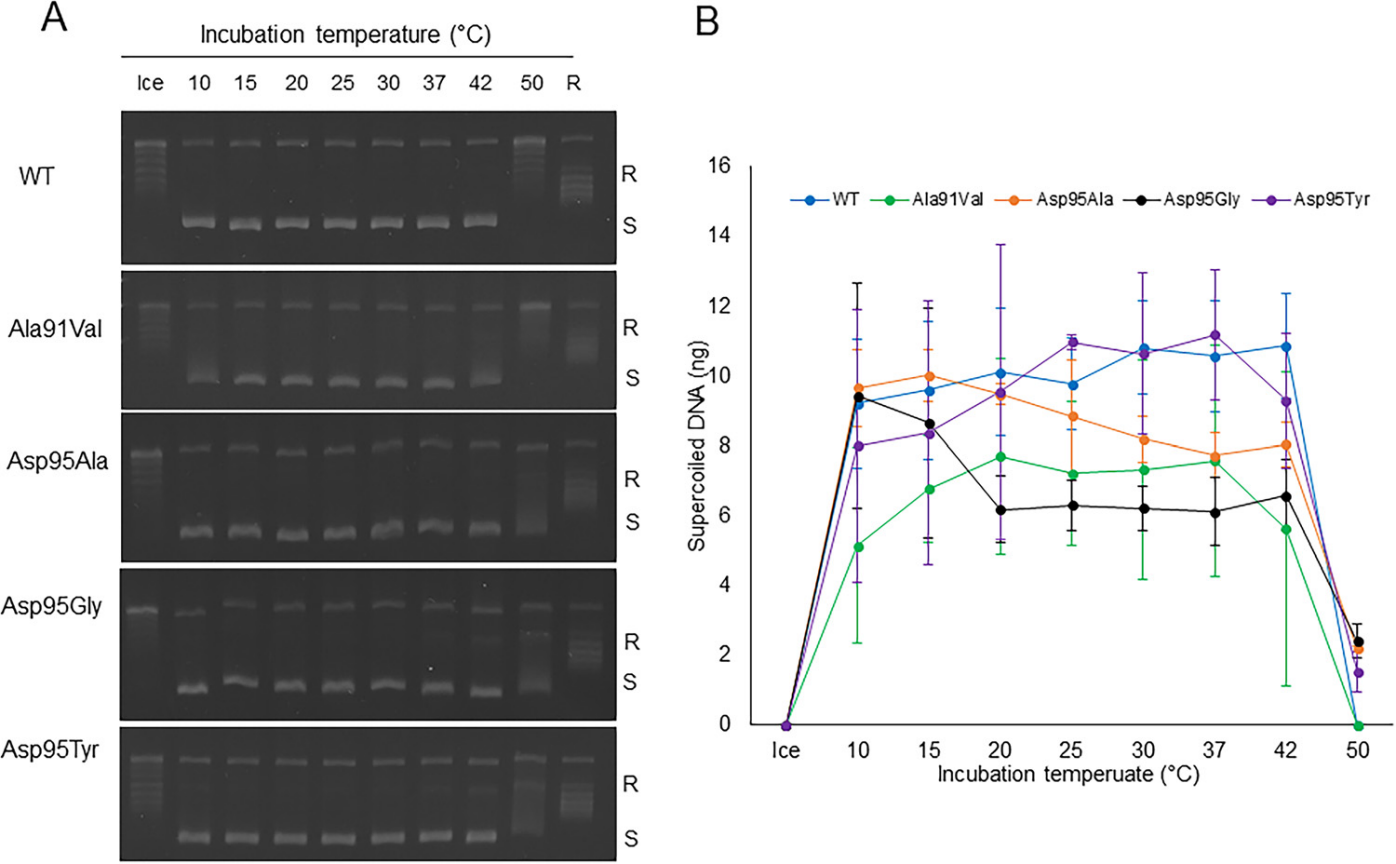
Temperature-dependent M. avium DNA gyrase supercoiling activities. Assays were performed by incubating the reaction mixture on ice and a range of temperatures from 10 to 50°C with WT or mutant GyrA and WT GyrB. (A) Gel electrophoresis of supercoiling activity. (B) Quantification of supercoiled DNA in corresponding DNA gyrase assay. R, relaxed DNA; S, supercoiled DNA.

The supercoiling activities were confirmed when GyrA, GyrB, and ATP were present; no supercoiling activity was observed in their absence (Fig. 2). We found that ≥5 nM of each DNA gyrase subunit (WT or mutant GyrA and GyrB) was enough to achieve a high rate of supercoiling activity (Fig. S2A and B) at 37°C and 60 min. Using a concentration of 7.5 nM, we tested the temperature-dependent supercoiling activity of WT and mutant GyrA and GyrB and found that high DNA gyrase supercoiling activity was obtained from 10°C to 42°C (Fig. 3A and B). Since we were interested to assess the inhibitory effects of FQs on DNA supercoiling activity in the human body, we decided to use the human body temperature of 37°C for our experiments.

### Inhibitory effects of FQs against M. avium WT and mutant DNA gyrase.

**FIG 4:**
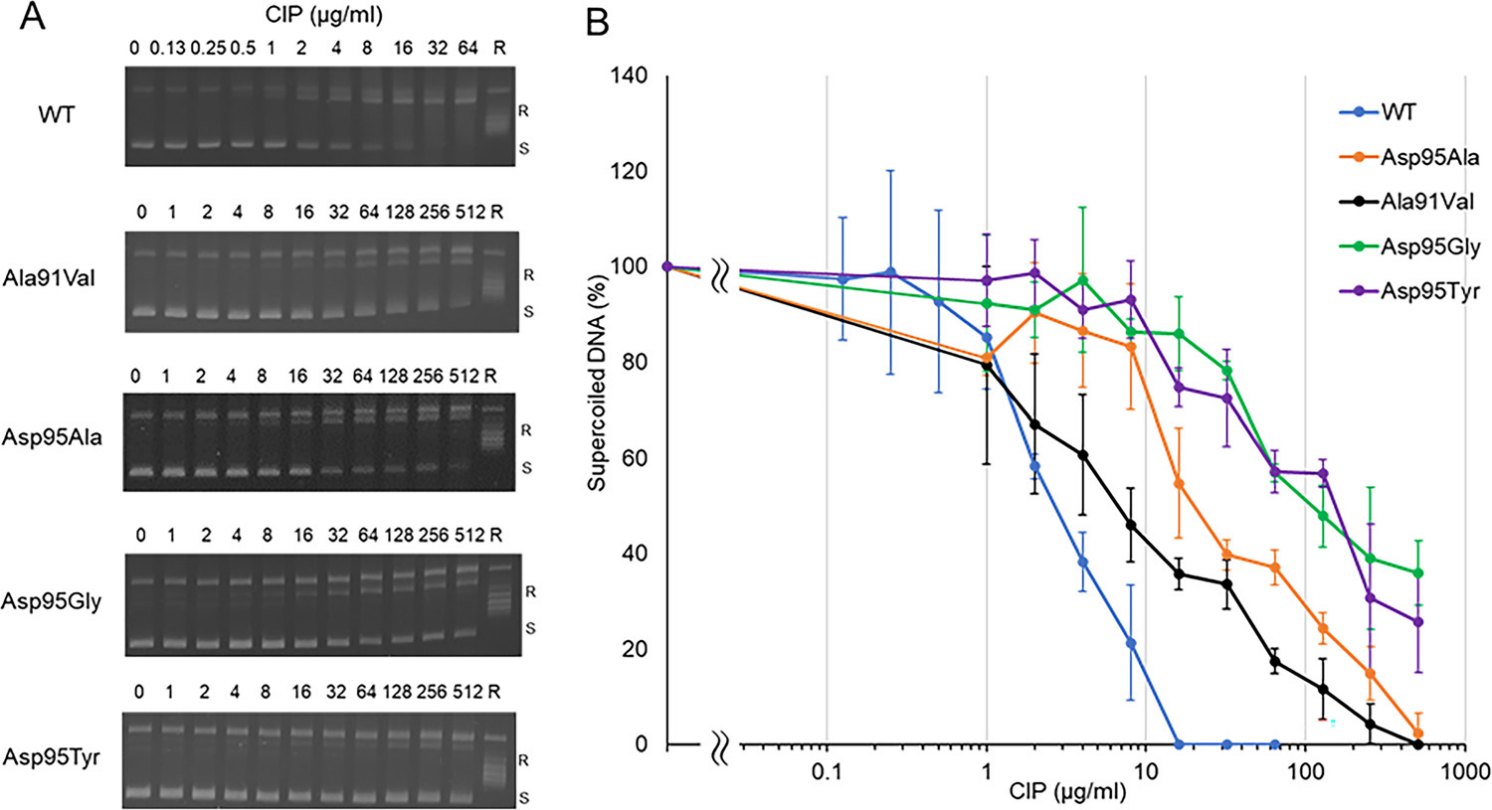
Inhibition of DNA supercoiling activity of M. avium DNA gyrase by ciprofloxacin (CIP). Inhibitory activity of CIP was examined against WT GyrA and four GyrA mutant proteins (Ala91Val, Asp95Ala, Asp95Gly, and Asp95Tyr). (A) Representative gel electrophoresis shows CIP concentration-dependent reduction of the amount of supercoiled DNA. (B) Quantification of amount of supercoiled DNA obtained from the CIP-induced supercoiling activity. R, relaxed DNA; S, supercoiled DNA. All assays were conducted at least in triplicate. Error bars represent the standard deviations (SD) of three independent experiments.

**FIG 5:**
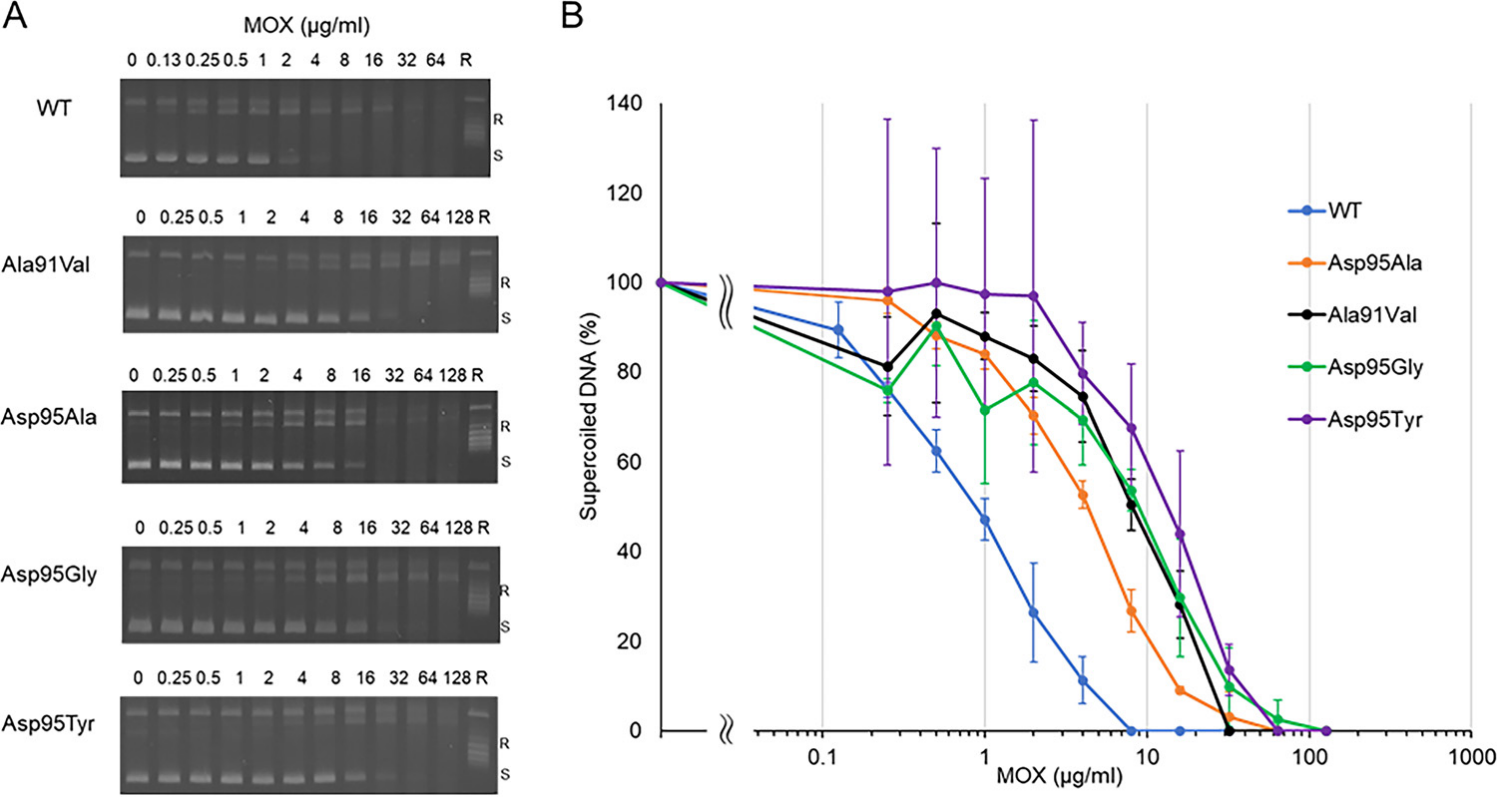
Inhibition of DNA supercoiling activity of M. avium DNA gyrase by moxifloxacin (MOX). Inhibitory activity of MOX was examined against WT GyrA and four GyrA mutant proteins (Ala91Val, Asp95Ala, Asp95Gly, and Asp95Tyr). (A) Representative gel electrophoresis shows CIP concentration-dependent reduction of the amount of supercoiled DNA. (B) Quantification of amount of supercoiled DNA obtained from the MOX-induced supercoiling activity. R, relaxed DNA; S, supercoiled DNA. All assays were conducted at least in triplicate. Error bars represent the SD of three independent experiments.

**FIG 6:**
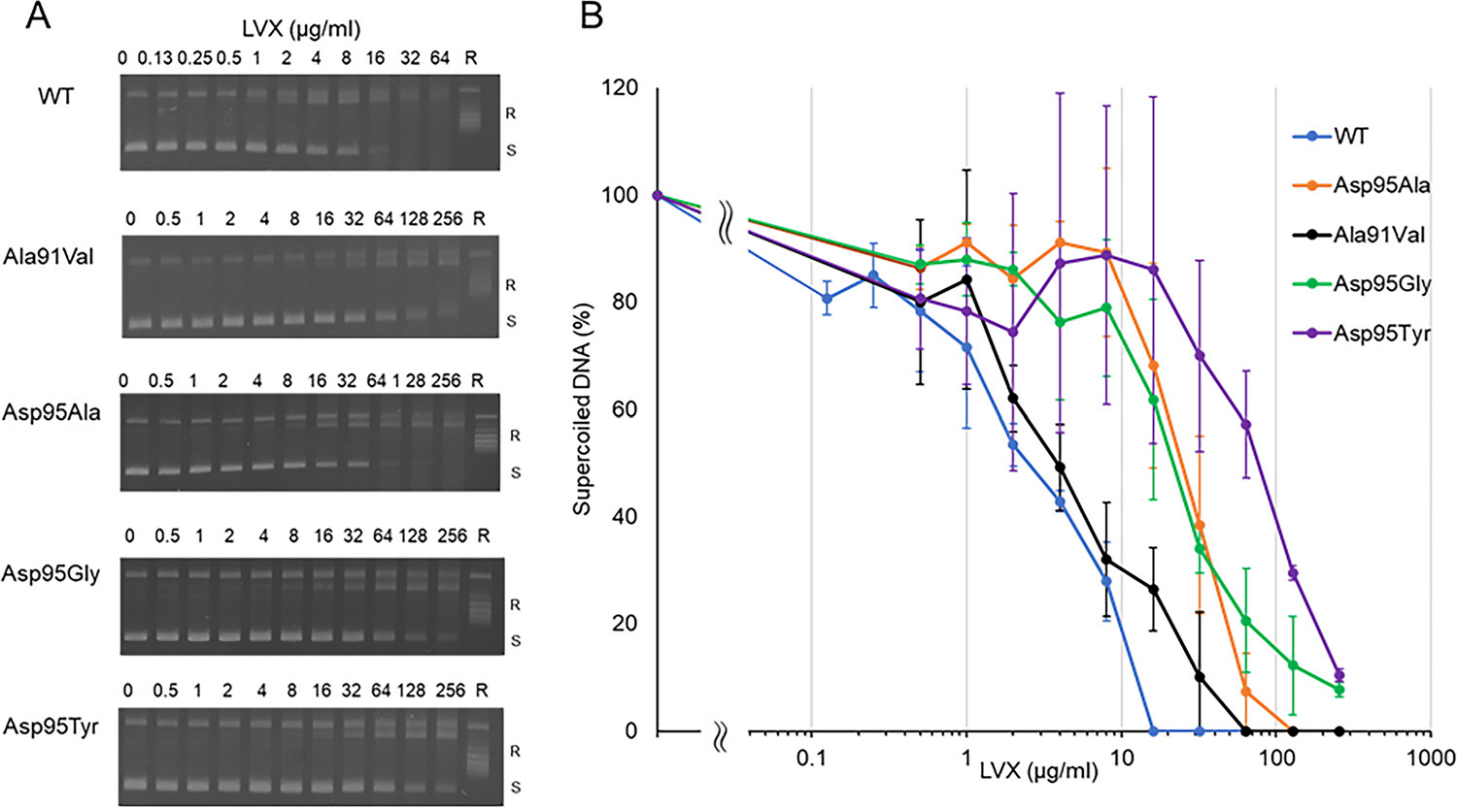
Inhibition of DNA supercoiling activity of M. avium DNA gyrase by levofloxacin (LVX). Inhibitory activity of LVX was examined against WT GyrA and four GyrA mutant proteins (Ala91Val, Asp95Ala, Asp95Gly, and Asp95Tyr). (A) Representative gel electrophoresis shows LVX concentration-dependent reduction of the amount of supercoiled DNA. (B) Quantification of amount of supercoiled DNA obtained from the LVX-induced supercoiling activity. R, relaxed DNA; S, supercoiled DNA. All assays were conducted at least in triplicate. Error bars represent the SD of three independent experiments.

The inhibitory effects of various concentrations of the FQs ciprofloxacin (CIP), MOX, and levofloxacin (LVX) against WT and mutant DNA gyrases, as revealed by the DNA supercoiling assay, are shown in Fig. 4, 5, and 6, and concentrations of each FQ required to inhibit the supercoiling activity by 50% (IC_50_) are shown in Table 1. Each FQ was tested at different highest concentrations based on their inhibitory effect (Fig. 4 to 6) against each gyrase. Each FQ showed dose-dependent inhibition of supercoiling activity, with IC_50_s ranging from 0.9 μg/mL with the MOX and WT GyrA combination to 106.8 μg/mL with the CIP and GyrA Asp95Tyr combination. While all the mutant GyrAs gave higher IC_50_ values than the WT, the GyrA Asp95Tyr mutant was highly resistant to inhibition by each FQ and gave the highest IC_50_s for each FQ tested (Table 1). MOX had the highest inhibitory effect, with an IC_50_ ranging from 0.9 to 13.5 μg/mL, and was the most effective FQ for Asp95 mutants, while CIP had the lowest inhibitory effect, with an IC_50_ ranging from 3 to 106.8 μg/mL. LVX showed highest effectiveness to the Ala91Val mutant (Table 1).

**TABLE 1:** IC_50_s of fluoroquinolone-inhibited DNA supercoiling activity

Fluoroquinolone	IC_50_ (μg/mL)				
	Wild type	Ala91Val	Asp95Ala	Asp95Gly	Asp95Tyr
Ciprofloxacin	3.0 ± 0.8	10.2 ± 0.3	33.4 ± 7.0	53.0 ± 4.7	106.8 ± 4.0
Moxifloxacin	0.9 ± 0.1	9.5 ± 0.2	4.2 ± 0.3	11.3 ± 3.5	13.5 ± 0.4
Levofloxacin	3.7 ± 0.4	5.0 ± 1.5	26.6 ± 5.9	23.3 ± 2.4	86.1 ± 6.0

### FQ susceptibility testing of WT and mutant clinical M. avium isolates.

The results of *in vitro* FQ susceptibility testing using the randomly selected eight WT and four *gyrA* mutant M. avium subsp. *hominissuis* clinical isolates collected in Japan are shown in Table 2. The clinical isolates with Asp95Gly and Asp95Tyr mutations in *gyrA* were highly resistant to FQs and had higher MIC values for MOX (≥4 μg/mL), LVX (≥8 μg/mL), and CIP (≥16 μg/mL) than the WT isolates for MOX (<0.125 to 1 μg/mL), LVX (<0.5 to 4 μg/mL), and CIP (<0.5 to 2 μg/mL) (Table 2).

**TABLE 2:** *In vitro* MICs of levofloxacin, ciprofloxacin, and moxifloxacin for 12 M. avium subsp. *hominissuis* clinical isolates

Sample ID	*gyrA* mutation	MIC (μg/mL) of:		
		Levofloxacin	Ciprofloxacin	Moxifloxacin
Koav 1	None	<0.5	<0.5	<0.125
Koav 2	None	2	<0.5	0.5
Koav 3	Asp95Gly	64	32	8
Koav 11	Asp95Tyr	8	16	4
Koav 13	Asp95Gly	128	64	16
Koav 15	None	4	2	0.5
Koav 16	None	<0.5	<0.5	<0.125
Koav 19	None	2	1	0.5
Koav 20	None	<0.5	<0.5	<0.125
Koav 21	None	4	2	1
Koav 25	None	4	2	1
CI-A2	Asp95Tyr	64	32	8

## DISCUSSION

To characterize M. avium DNA gyrase activity and elucidate the impact of quinolone-associated amino acid substitutions in FQ resistance, we focused on amino acid substitutions Ala91Val, Asp95Ala, Asp95Gly, and Asp95Tyr in GyrA. Unlike previous studies (13, 15) where FQ-resistant clinical M. avium did not have any mutation in the QRDR of *gyrA*, our study detected four FQ-resistant isolates with *gyrA* QRDR mutations. These four mutant isolates with two types of amino acid substitutions (Asp95Gly and Asp95Tyr) in two isolates each were found to be MOX resistant in the MIC assay and were determined to be FQ resistant, as defined by CLSI guidelines (32). The MICs of FQs for the Asp95Gly mutant were at least 8- to 16-fold higher than those for WT isolates for MOX, at least 16- to 32-fold higher for CIP, and at least 16- to 32-fold higher for LVX. Similarly, the MICs of FQs for the Asp95Tyr mutant were 4- to 8-fold higher than those for WT isolates for MOX, 8- to 16-fold higher for CIP, and 2- to 16-fold higher for LVX (Table 2). These findings suggested that, as in M. tuberculosis (19, 22, 27), mutations in the QRDR of *gyrA* in M. avium significantly contribute to FQ resistance. The amino acid substitution Ala91Val was selected for further analysis, as this mutation had spontaneously emerged *in vitro* when M. avium was grown under FQ selective pressure, as previously described (25). This mutant isolate was highly resistant to ofloxacin, with an MIC of 128 μg/mL, compared to that for the WT (16 μg/mL) (25).

Similar to FQ-resistant MAC isolates, FQ-resistant clinical isolates of Mycobacterium abscessus, another important causative agent of NTM infections, have also been reported to lack mutations in the QRDR of GyrA (33). However, in a previous study from Brazil that investigated GyrA substitutions in CIP-resistant members of the M. abscessus complex, the Ala91Val substitution was observed in 89% (31/35 isolates) of Mycobacterium massiliense, Asp95Gly was found in one M. abscessus isolate, while a double amino acid substitution of Ala91Val and Asp95Asn was uncovered in an Mycobacterium chelonae isolate (26). These mutations were homologous to the mutations in the QRDR of MAC described in this study, indicating that these amino acid substitutions in the GyrA QRDR impact FQ resistance in NTM bacteria, including M. avium.

To the best of our knowledge, there is only one study that has reported the DNA gyrase supercoiling activity of M. avium (27), where the supercoiling activity of WT DNA gyrase was examined without detailed characterization of DNA gyrase activity. Here, we have characterized both WT and mutant DNA gyrases in terms of quality, quantity, incubation temperature, and time course activity (Fig. S1 and S2; Fig. 2). Although, the previous study (34) incubated the reaction mixture at 30°C, our results showed that M. avium DNA gyrase has a wide range of stable supercoiling activity from 10°C to 42°C, indicating the adaptability of M. avium across different environments and hosts, ranging from an outdoor environment to an avian host (Fig. 3A and B). Furthermore, a previous study showed that gyrase activity in Mycobacterium leprae was most effective at 30°C compared to 37°C, suggesting that the optimum temperature for gyrase activity depends on the natural ecology of each bacterium (35).

In the supercoiling assay, the IC_50_ of FQs against mutant DNA gyrases were 1.3- to 35.6-fold higher than those against WT enzymes. The Asp95Tyr was the most resistant mutant, with an IC_50_ 15- to 35.6-higher than for the WT, followed by the Asp95Gly mutant, for which the IC_50_ was 12.5- to 17.6-fold higher than for the WT. This finding confirmed that these amino acid substitutions significantly contribute to FQ resistance, as previously shown in M. tuberculosis (21), where Ala90Val and Asp94Gly showed 17.5- and 25-fold-higher resistance than WT, as well as in a previous study (22), where the biochemical basis for the FQ resistance caused by these mutations was revealed.

When considering the inhibitory activity of FQs against mutant DNA gyrases, CIP and LVX had the highest inhibitory activity against DNA gyrase with the Ala91Val substitution, and MOX had the highest inhibitory activity against DNA gyrase with the Asp95Ala substitution; all FQs had the lowest inhibitory activity against DNA gyrase with the Asp95Tyr substitution. There was a slight difference between FQ resistance and the mutation profile between MICs and supercoiling assays, as the MIC assay showed that isolates with the Asp95Gly substitution had up to a 2-fold-higher MIC than did the Asp95Tyr mutant with all tested FQs, whereas the DNA supercoiling assay showed that, relative to the Asp95Gly substitution, the Asp95Tyr substitution conferred 2-fold-higher resistance against CIP, 1.1-fold higher against MOX, and 3.6-fold higher against LVX. The difference in the way the drug-enzyme-DNA complex interacts between the supercoiling assay and MIC assay could be a contributing factor to the observed discrepancy. In summary, our results showed that amino acid substitutions of Asp95Gly and Asp95Tyr confer high-level resistance against FQs in M. avium, suggesting that M. avium strains with a mutation in the QRDR of GyrA are FQ resistant. The substitution of Asp95Tyr introduces a bulky hydrophobic side chain to confer high-level FQ resistance, most likely by reducing FQ binding to the gyrase-DNA complex and increasing the distance between the catalytic residue of the enzyme and the DNA bases adjacent to the cleavage site (22, 30, 36). Our supercoiling assay confirmed that, similar to M. tuberculosis, mutations in QRDR of *gyrA* also strongly contributed to FQ resistance in M. avium. Furthermore, our MIC experiments confirmed that M. avium strains with FQ-associated mutation in QRDR of GyrA are resistant to FQs.

However, we note that many FQ-resistant clinical M. avium isolates do not have mutations in the QRDR of *gyrA* (13, 15), indicating that other mechanisms are also involved in FQ resistance. Efflux pumps have been suggested to confer FQ resistance in NTM (37). Induction of efflux pump activity upon drug exposure to reduce the intracellular concentration of a drug has been found to be a general first step in the evolution of mycobacterial drug resistance (38). A combination of clarithromycin and efflux pump inhibitors has been shown to significantly decrease the MIC of clarithromycin (39). While our *in vitro* DNA gyrase assay showed that the mutant M. avium DNA gyrases significantly contributed to FQ resistance, the common clinical scenario of FQ resistance in the absence of mutations in M. avium DNA gyrase genes may be due to induction of efflux pumps which expel FQ and reduce interactions of FQ and DNA gyrase. Mutations in the *gyrB* of E. coli (40) and M. tuberculosis H37Ra (41) have been found to contribute FQ resistance. A recent study reported a low frequency of FQ-resistant clinical M. tuberculosis isolates with *gyrB* mutations (42), suggesting a potential role of *gyrB* mutation on FQ resistance. Although we did not find any mutations in *gyrB* of the clinical isolates in this study, the potential role of mutation in GyrB of M. avium should be considered. There may be other unknown mechanisms for FQ resistance in M. avium. Thus, the detailed molecular mechanism of FQ resistance in M. avium needs further exploration.

We found that MOX was the most effective FQ for M. avium. It had the lowest MICs, <0.125 to 1 μg/mL and 4 to 16 μg/mL for WT and mutant isolates, respectively (Table 2). The superiority of MOX was further supported by results of the supercoiling assay, which showed the lowest IC_50_s both for WT and mutant gyrases. The IC_50_ of MOX against the GyrA with the Asp95Tyr substitution, the mutant enzyme with the highest resistance against FQs, was 7.9-fold and 6.3-fold lower than those for CIP and LVX, respectively (Table 1). MOX has been recommended for MAC infections (11), and MOX-containing regimens have improved the treatment outcome of MAC (including M. avium) lung disease (43). The structural differences between MOX, CIP, and LVX are the substitutions at positions 1, 7, and 8: MOX has a cyclopropyl group at R1, an azabicyclo group at R7, and a methyl group at R8; CIP also has a cyclpropyl group at R1, a simple pipeazine group at R7, and no substitution at R8; whereas LVX has a bridge at R1-R8 and an *N*-methyl piperazine at R7 (Fig. S4). The R7 position directly interacts with DNA gyrase, so the bulkier azabicyclo group at R7 of MOX may enhance the interaction of MOX with GyrA (44). Furthermore, the bulkiness of MOX at R7 reduces expulsion via efflux proteins in Streptococcus pneumoniae and hence a higher potency via higher cellular accumulation (45). In a previous study with M. tuberculosis gyrases (22), MOX was found to maintain higher inhibitory activity against WT and mutant gyrases than CIP by its ability to form a stable binding complex with enzyme. This stable FQ-gyrase-DNA complex was further improved by introducing an 8-methyl derivative of MOX (22). Thus, MOX may have a greater inhibitory activity against M. avium because of its increased interaction with DNA gyrase and reduced efflux. Structural modifications of FQs, especially around positions 1, 7, and 8, have been shown to be effective against mutant gyrases in different bacteria (46–49). Thus, the development of new FQs with a similar structure to MOX (22), or with a higher intracellular accumulation and efficient interaction with the DNA-DNA gyrase complex, have the potential to be effective candidates for the treatment of M. avium-associated NTM infections. We note that LVX had 2-fold-higher inhibitory activity than MOX and CIP on the Ala91Val mutant. However, we do not have MIC data for this mutant, so the effectiveness of LVX on the Ala91Val mutation should be further explored.

In summary, our study confirmed that amino acid substitutions of Ala91Val, Asp95Ala, Asp95Gly, and Asp95Tyr in the QRDR of GyrA of M. avium strongly contribute to FQ resistance, similar to what has been observed in M. tuberculosis. Clinical M. avium strains with FQ-associated mutations in the QRDR of GyrA were found to be resistant to FQs. However, since many FQ-resistant M. avium isolates do not have these mutations, further exploration of other FQ resistance mechanisms is needed.

## MATERIALS AND METHODS

### Materials.

FQs, CIP, LVX, and MOX that were used in inhibition assays were purchased from LKT Laboratories, Inc. (St. Paul, MN, USA). Kanamycin and ampicillin were purchased from Fujifilm Wako Pure Chemical Co., Ltd. (Osaka, Japan). Restriction enzymes and lambda DNA-HindIII DNA marker were obtained from New England Biolabs, Inc. (Ipswich, MA). DNA ligation kit, Mighty Mix, and Mighty TA cloning kit were purchased from TaKaRa Bio Inc. (Shiga, Japan). Relaxed pBR322 DNA was purchased from John Innes Enterprises Ltd. (Norwich, United Kingdom). Luria-Bertani (LB) broth (Lennox) and LB agar were purchased from Sigma (St. Louis, MO, USA). Agarose S was purchased from Nippon Gene (Toyoma, Japan). Agarose I was obtained from Dojindo (Kumamoto, Japan). Gel red was obtained from Fujifilm Wako Pure Chemical Co., Ltd. (Osaka, Japan).

### Bacterial strains and plasmids.

The pMD20-T (TaKaRa Bio Inc., Shiga, Japan) was used to construct a cloning vector, whereas pET29a+ plasmid (Merck KGaA, Darmstadt, Germany) was used to construct an expression vector to produce WT and mutant GyrA proteins. Escherichia coli DH5α (TaKaRa Bio Inc., Shiga Japan) was used as a host for cloning. E. coli Rosetta-gami 2(DE3) pLysS (Merck KGaA, Darmstadt, Germany) was used for protein expression. Table 2 provides information on the eight WT and four *gyrA* mutant clinical strains of M. avium subsp. *hominissuis* isolated from Japan that were used for the MIC assay.

### Construction of M. avium DNA gyrase expression plasmids.

Genomic DNA of M. avium subsp. *hominissuis* strain (HP 59) isolated in Hokkaido, Japan, was used to amplify *gyrA* and *gyrB* via PCR. The PCR mixture (25 μL) consisted of 1× LA PCR buffer (Mg^2+^ free), 2.5 mM MgCl_2_, 0.4 mM each deoxynucleoside triphosphate, 0.4 μM each primer, 1.25 units of TaKaRa LA *Taq* (TaKaRa Bio Inc.), and 2.5 ng of genomic DNA. The primer information is shown in Table S1. The PCR thermal cycle consisted of initial denaturation at 98°C for 2 min, 35 cycles of denaturation at 98°C for 5 s, annealing at 60°C for 5 s, and extension at 72°C for 30 s to 3 min (depending upon the size of PCR products), and a final extension at 72°C for 5 min. PCR products were purified by ethanol precipitation using sodium acetate or by using Wizard SV gel and the PCR Cleanup system (Promega, Madison, WI, USA). Figure S3 outlines the procedure for the construction of DNA gyrase-expressing vectors. The ligation product was transformed into E. coli DH5α and plated onto LB agar containing either ampicillin (100 μg/mL) or kanamycin (50 μg/mL). Colonies were selected and expanded in LB broth, and plasmids were purified using a Wizard Plus SV Minipreps DNA purification system (Promega, Madison, WI, USA). Nucleotide sequencing was performed using the BigDye Terminator (version 3.1) cycle sequencing kit and an ABI Prism 3130x genetic analyzer (Applied Biosystems). BioEdit software (version 7.2.5.0) was used to confirm the sequences of DNA gyrase genes in the plasmids.

### Expression and purification of recombinant M. avium DNA gyrase subunits.

Recombinant M. avium DNA gyrase subunits were expressed and purified as previously described (46–49) with minor modifications. Briefly, each recombinant plasmid containing *gyrA* (WT and mutant) and *gyrB* was transformed into E. coli Rosetta-gami 2(DE3) pLysS. Single colonies were picked and cultured overnight in 10 mL LB broth containing 50 μg/mL kanamycin. Overnight cultures were then inoculated into 500 mL of LB broth containing 50 μg/mL kanamycin at 1:100 dilution. Cells were cultured at 37°C by shaking until the optical density (OD) at 590 reached 0.4 to 0.6. Expression of gyrase subunits was induced by the addition of 1 mM isopropyl beta-d-thiogalactopyranoside (Wako Pure Chemical Industries Ltd., Osaka, Japan) and further incubation at 18°C for 16 to 18 h. The harvested E. coli cells in 1× native binding buffer (50 mM sodium phosphate, 500 mM NaCl; pH 7.4 to 8) containing EDTA-free protease inhibitor cocktail (Roche, Mannheim, Germany) were sonicated on ice (10 times, 40 s of sonication, 40 s of cooling, duty cycle of 30%, and output of 4%) by using a Sonifier 250 (Branson, Danbury, CT), and the supernatant of the sonicated lysate was purified by Ni-nitrilotriacetic acid agarose (Invitrogen, CA, USA) columns. The columns were washed with 1× native binding buffer containing 60 mM imidazole (Wako Pure Chemical Industries Ltd., Osaka, Japan) and finally eluted with elution buffer containing 1× native binding buffer with 250 mM imidazole. The eluted proteins were concentrated with an Amicon Ultra 15, 30 kDa system (Millipore, Billerica, MA, USA), and imidazole was removed by buffer exchange using a PD-10 column (Cytiva, United Kingdom) to DNA gyrase dilution buffer (50 mM Tris-HCl [pH 7.5], 100 mM KCl, 2 mM dithiothreitol [DTT], 1 mM EDTA). The eluted fractions of the final purified protein were stored in 40% glycerol at −80°C until further use. A representative sample of the protein was collected at different stages of the protein expression and purification procedures, and the quality and quantity of the purified protein were analyzed using SDS-PAGE.

### DNA supercoiling activities and inhibition by FQs.

A combination of purified GyrA and GyrB subunits was used to determine DNA supercoiling activity as previously described (46–49). Briefly, the reaction mixture of 30 μL consisted of DNA gyrase assay buffer (35 mM Tris-HCl [pH 7.5], 24 mM KCl, 4 mM MgCl_2_, 2 mM DTT, 1.8 mM spermidine, 6.5% glycerol, and 0.1 mg/mL bovine serum albumin), 1 mM ATP, relaxed pBR322 DNA (1.5 nM), and purified GyrA and GyrB subunits (7.5 nM each). The mixture was incubated at 37°C for 60 min and stopped by adding 8 μL of 5× dye mix (5% SDS, 25% glycerol, and 0.25 mg/mL bromophenol blue). Next, 5 to 10 μL of each reaction mixture was subjected to electrophoresis in a 1% agarose-I gel in 1× Tris-acetate-EDTA buffer at 50 mA for 96 min. The gels were stained with 1× gel red (Wako Pure Chemical Industries Ltd., Osaka, Japan) for 30 min and visualized with a FAS-Digi transilluminator (Nippon Genetics, Tokyo, Japan). Supercoiling activity was assessed by measuring the brightness of the supercoiled DNA band using ImageJ software (https://imagej.nih.gov/ij/). The roles of ATP and M. avium gyrase subunits in DNA supercoiling activity were confirmed similarly to the above reaction mixture by using different combinations of ATP, GyrA, and GyrB subunits. A concentration-dependent supercoiling assay using 0.25 to 12.5 nM GyrA and GyrB was conducted to optimize the concentration of DNA gyrase. Similarly, temperature-dependent DNA gyrase activity was performed on ice at 10, 15, 20, 25, 30, 37, 42, and 50°C to check the enzymatic activity and identify the optimal temperature for the assay. The inhibitory effects of FQs, CIP, MOX, and LVX on DNA gyrase activity were assessed by determining the FQ concentration required to inhibit the supercoiling activity by 50% (IC_50_). Band intensity data corresponding to supercoiling activity were uploaded into an IC_50_ calculator (https://www.aatbio.com/tools/ic50-calculator). All assays were conducted at least in triplicate on the same day to confirm reproducibility.

### FQs susceptibility testing of WT and mutant clinical M. avium isolates.

To correlate the IC_50_ data obtained for FQ-dependent inhibition of DNA supercoiling activity with phenotypic drug susceptibility, a MIC assay was performed on 12 preserved M. avium subsp. *hominissuis* clinical isolates (8 *gyrA* WT, 2 *gyrA* Asp95Gly, and 2 *gyrA* Asp95Tyr) (Table 2). MIC testing on clinical strains with Ala91Val and Asp95Ala mutations could not be performed, as these mutations were not found in any of the clinical strains analyzed. MICs were determined using the broth microdilution method in 7H9 medium (Difco Middlebrook, Sparks, MD, USA) supplemented with 10% oleic acid-albumin-dextrose-catalase and 0.5% Tween 80 (Wako Fujifilm, Osaka, Japan). The frozen stocks of M. avium were inoculated in 2% Ogawa medium (Serotec, Sapporo, Japan). Next, the solid culture was transferred into 4 mL of 7H9 broth and cultured until an OD at 590 nM of 0.14 to 0.16 was reached. This culture was further diluted 40 times and used as a starting culture for the MIC assay. The assay was carried out in a 96-well round bottom culture plate in a final volume of 200 μL with 100 μL of starting culture and 100 μL of drug, with or without dilution in 7H9 broth. The outer wells of the plate were filled with 200 μL sterile distilled water. Each plate had two medium-only controls, a drug-free control, and a positive control with kanamycin at 25 μg/mL. The three FQs, CIP, MOX, and LVX, that were used in the DNA gyrase assay were used in the MIC assay. The plates were sealed with plastic membranes, placed in a container with moist cotton, and incubated at 37°C for 14 days. Each experiment was performed at least in duplicate. The culture was monitored on days 0, 1, 7, 10, and 14 by taking a picture. On day 14, MICs were determined as the lowest FQ concentration that inhibited visible bacterial growth, which was confirmed by at least three investigators.
