# Supplementary material for: Characterization of DNA Gyrase Activity and Elucidation of the Impact of Amino Acid Substitution in GyrA on Fluoroquinolone Resistance in Mycobacterium avium
Source: Microbiol Spectr. 2023 Apr 17;11(3):e05088-22. doi: 10.1128/spectrum.05088-22 (PMC10269562; doi:10.1128/spectrum.05088-22)
Supplement: Supplemental file 1 — Supplemental material. Download spectrum.05088-22-s0001.pdf, PDF file, 1.2 MB [file spectrum.05088-22-s0001.pdf]

Table S1. Description of primers used in this study

ID	Primer name	Target	Sequence (5-->3)	Use	Remarks
ON-1	Mav-NdeI-H6-gyrA5	*gyrA*	AAA*CATATG*CACCATCACCATCACCACATGACTGACACCACGCTGCCAC	PCR	N-ter His-Tag
ON-2	Mav-gyrA3-HindIII R	*gyrA*	AAA*AAGCTT*CTAGCCGTCCGACCCCGCGGCACCG	PCR	C-ter
ON-3	Mav-gyrA 346-367	*gyrA*	CAATTTTGGTTCGCCGGGCAACG	Sequencing	
ON-4	Mav-gyrA 762-781	*gyrA*	GCGCGGAGTCGTTGAGGTGG	Sequencing	
ON-5	Mav-gyrA 1165-1186	*gyrA*	CCCACATCCTGCGCGGTCTG	Sequencing	
ON-6	Mav-gyrA 1587-1608	*gyrA*	CACCAAGACCGACCTGTACCGC	Sequencing	
ON-7	Mav-gyrA 2003-2023	*gyrA*	CCGAGGACGATCTGCTGCTGG	Sequencing	
ON-8	Mav-gyrA-A90V_F	*gyrA*	CGCACGGCGACG<u>**T**</u>CTCGATCTACGA	PCR	For mutation
ON-9	Mav-gyrA-A90V_R	*gyrA*	TCGTAGATCGAG<u>**A**</u>CGTCGCCGTGCG	PCR	For mutation
ON-10	Mav-gyrA-D94A_F	*gyrA*	CCTCGATCTACG<u>**C**</u>CACCCTGGTGCG	PCR	For mutation
ON-11	Mav-gyrA-D94A_R	*gyrA*	CGCACCAGGGTG<u>**G**</u>CGTAGATCGAGG	PCR	For mutation
ON-12	Mav-gyrA-D94G_F	*gyrA*	CCTCGATCTACG<u>**G**</u>CACCCTGGTGCG	PCR	For mutation
ON-13	Mav-gyrA-D94G_R	*gyrA*	CGCACCAGGGTG<u>**C**</u>CGTAGATCGAGG	PCR	For mutation
ON-14	Mav-gyrA-D94Y_F	*gyrA*	GCCTCGATCTAT<u>**T**</u>ACACCCTGGTGC	PCR	For mutation
ON-15	Mav-gyrA-D94Y_R	*gyrA*	GCACCAGGGTGT<u>**A**</u>ATAGATCGAGGC	PCR	For mutation
ON-16	Mav-gyrA 479-498R	*gyrA*	CACCGTCGGCTCTTGCACCC	PCR	For mutation
ON-17	Mav-NdeI-gyrB5	*gyrB*	AAA*CATATG*GCTGCCCAGAAGAAGAAGGC	PCR	N-ter
ON-18	Mav-gyrB3-H6-HindIII R	*gyrB*	AAA*AAGCTT*TTAATGGTGATGGTGATGGTGAACGTCTAGGAAGCGAACGTC	PCR	C-ter His-Tag
ON-19	Mav-gyrB 332-354	*gyrB*	GCGAGAACAGCGGCTACAACGTC	Sequencing	
ON-20	Mav-gyrB 748-768	*gyrB*	CACCGCACCTTCCACTACCCC	Sequencing	
ON-21	Mav-gyrB 1134-1156	*gyrB*	CAACACCGAGGTGAAGTCGTTCG	Sequencing	
ON-22	Mav-gyrB 1577-1599	*gyrB*	ACCACAAGATCGTGTTGATGGCC	Sequencing	

Bold and underlined: mismatch bases

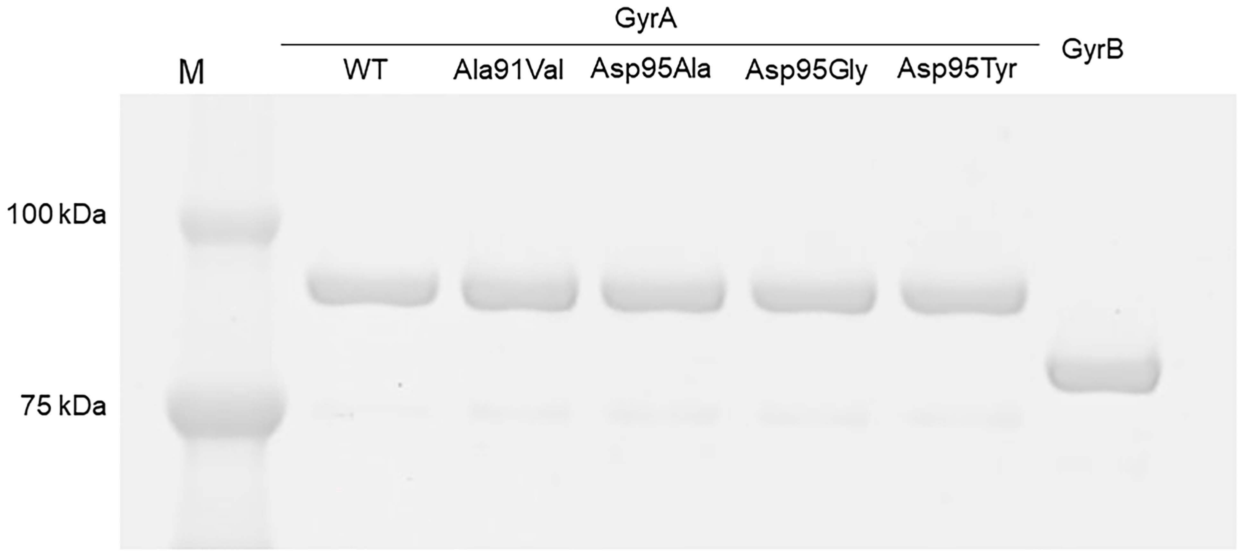

Fig. S1. SDS-PAGE analysis of purified recombinant *M. avium* DNA gyrase subunits. Three hundred ng of each recombinant DNA gyrase subunits were loaded into a SuperSep (TM) Ace, 5-20%, 17 well gel (Fujiflim Wako, Osaka, Japan) for electrophoresis.

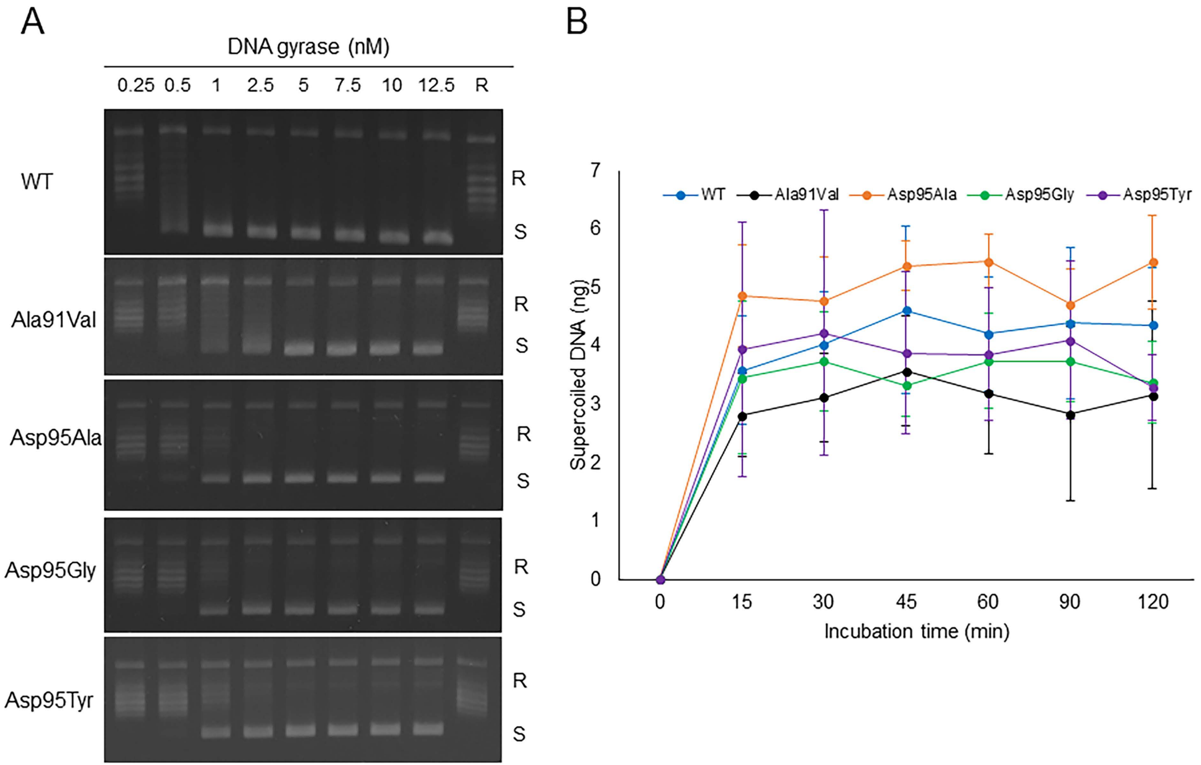

Fig S2. Optimization of concentration of DNA gyrase subunits. Concentration ranging from 0.25 nM to 12.5 nM of each DNA gyrase subunits WT-GyrA, Ala91Val-GyrA, Asp95Ala-GyrA, Asp95Gly-GyrA or Asp95Tyr-GyrA and WT-GyrB were used to assess optimal supercoiling activity. Assays were performed in triplicates. (A) Gel electrophoresis of supercoiling activity. (B) Quantification of supercoiled DNA in corresponding DNA gyrase assay. R: relaxed DNA; S: supercoiled DNA.

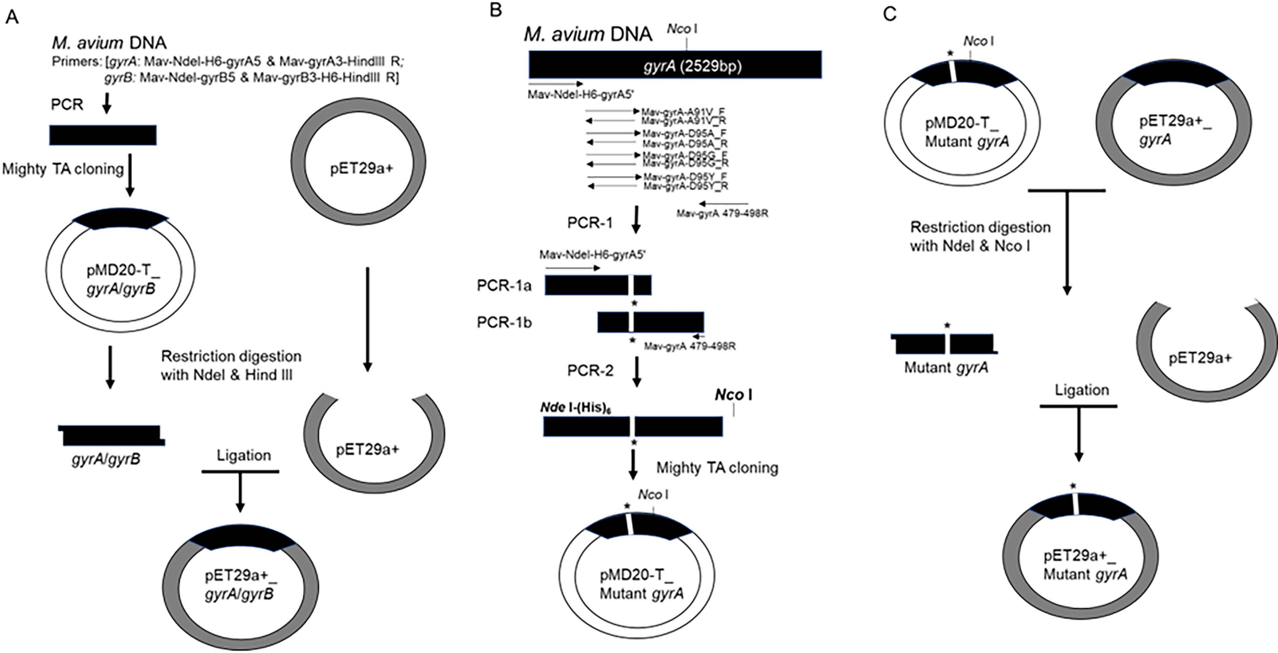

Fig S3. Strategy for construction of WT and mutant *M. avium* DNA gyrase expressing plasmid. (A) Construction of WT gyrA expressing plasmid, pET29a+_*gyrA*/*B*. WT *gyrA*/*B* was amplified by PCR with primers containing His-tag and restriction sites (NdeI and HindIII) (Table S1). The PCR product was ligated with cloning TA vector, pMD20-T. Both cloning vectors containing *gyrA*/*B* and expression vector pET29a+ were digested by NdeI and HindIII, and the corresponding digested product was ligated to construct the expressing plasmid pET29a+_gyrA/B. (B) Construction of mutant gyrA expression plasmid. Two PCRs were performed to amplify *gyrA* segments with a forward primer containing NdeI restriction site and a reverse primer containing mutant nucleotide substitution (PCR-1a) and a forward primer containing mutant nucleotide substitution and a reverse primer (PCR-1b), respectively (Table S1). The PCR-2 was performed using the DNA templates from PCR-1a and PCR-1b with the forward primer containing NdeI restriction site and the reverse primer. This PCR product containing NdeI and NcoI restriction site and mutant gyrA was cloned into pMD20-T. (C) Construction of expression vector containing mutant gyrA. The cloning vector pMD20-T containing mutant gyrA and expression vector containing WT gyrA were digested with NdeI and NCoI, and the corresponding segments were then ligated to construct expressing vectors pET29a+_*gyrA* carrying Ala91Val, Asp95Ala, Asp95Gly, and Asp95Tyr mutation, respectively.

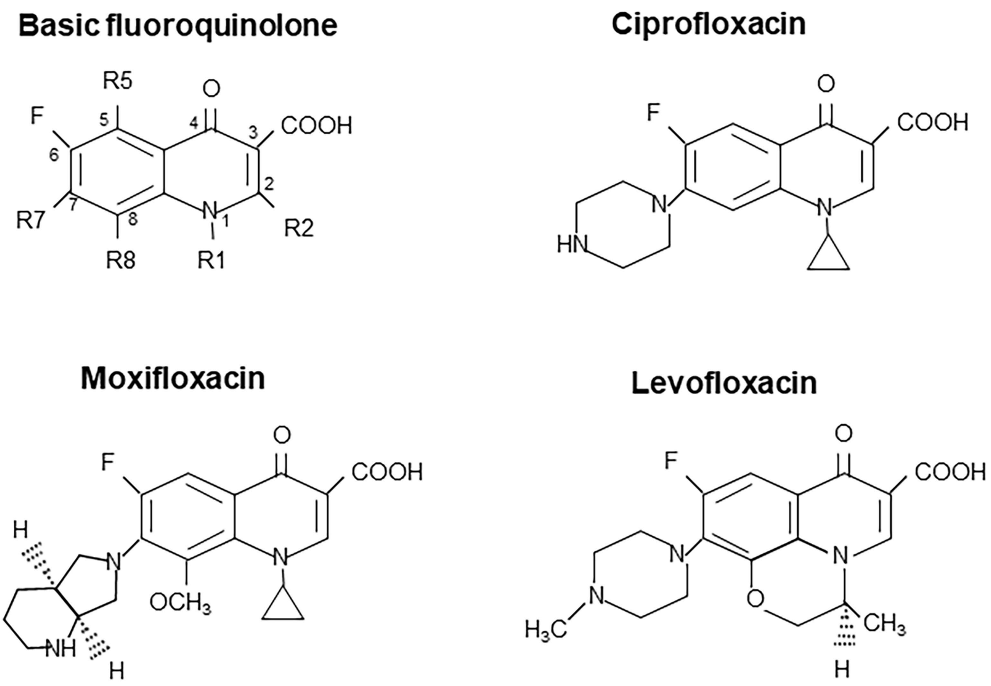

Fig. S4. Chemical structures of fluoroquinolones used in this study.
